# Surgical interventions in Severe Osteoarthritis: Pros and Cons

**DOI:** 10.26502/josm.511500192

**Published:** 2025-03-31

**Authors:** Andre Aabedi, Marcel P Fraix, Devendra K. Agrawal

**Affiliations:** 1 Departments of Translational Research, College of Osteopathic Medicine of the Pacific, Western University of Health Sciences, Pomona, California 91766 USA; 2 Physical Medicine and Rehabilitation, College of Osteopathic Medicine of the Pacific, Western University of Health Sciences, Pomona, California 91766 USA

**Keywords:** Arthritis, Arthrodesis, Arthroscopy, High tibial osteotomy, Osteoarthritis, Osteotomy, Pain, Surgical interventions, Total hip arthroplasty, Total joint arthroplasty, Total knee arthroplasty, Unicompartmental knee arthroplasty

## Abstract

Severe osteoarthritis (OA) is a debilitating condition that often necessitates surgical intervention when conservative treatments fail. We carefully reviewed the literature on the pros and cons of surgical options for severe OA, focusing on total joint arthroplasty (TJA) and other surgical techniques. Total joint arthroplasty, including total knee arthroplasty (TKA) and total hip arthroplasty (THA), is the most established surgical option for severe OA, providing significant pain relief, functional restoration, and improved quality of life. The American College of Rheumatology and the American Association of Hip and Knee Surgeons recommend proceeding to TJA without delay in patients with symptomatic moderate-to-severe OA unresponsive to nonoperative therapy. Osteotomies and cartilage repair procedures are less commonly performed and have limited evidence supporting their long-term efficacy in reducing OA progression. Arthroscopic interventions, such as lavage and debridement, do not alter disease progression and are not recommended for routine treatment of OA. While TJA is highly effective, it is associated with risks such as postoperative complications, revisions, and reoperations. The cost-effectiveness of TJA is well-documented, making it a favorable option for managing end stage OA. However, patient selection is crucial, and factors such as age, comorbidities, and obesity must be considered to optimize outcomes. Total joint arthroplasty remains the gold standard for surgical management of severe OA, offering substantial benefits in pain relief and functional improvement. Other surgical options, such as osteotomies and arthroscopy, have limited roles and should be considered based on individual patient factors and disease severity. Evidence-based guidelines support the timely use of TJA to enhance patient outcomes and quality of life.

## 1. Introduction

Osteoarthritis (OA) is a prevalent degenerative joint disease characterized by the progressive deterioration of articular cartilage and subchondral bone, leading to pain, stiffness, and functional impairment [1]. It affects over 240 million people globally, with significant impacts on quality of life, particularly in older adults [1]. The pathophysiology of OA involves complex interactions between mechanical, inflammatory, and metabolic factors, resulting in cartilage degradation, osteophyte formation, and synovial inflammation [2–6]. Many inflammatory mediators, including IL-33, IL-37, damage-associated molecular patterns, have been implicated in the underlying pathogenesis [2–5]. As OA progresses to severe stages, patients experience increased pain and disability, often necessitating surgical intervention [6,7].

There are several medicines that may provide relief from pain and reduce inflammation. These include acetaminophen, non-steroidal anti-inflammatory drugs, corticosteroids, duloxetine, and adjunct therapies like vitamin D and medicinal plants [8–10]. However, the effect of conservative treatments, like physical therapy, pharmacotherapy, and lifestyle modifications, are temporary and fail to provide adequate symptom relief [1–4]. Thus, the conservative treatments have limitations, including insufficient long-term efficacy and potential side effects from chronic medication use [1–10]. There is no effective disease-modifying therapy.

Surgical interventions are considered when conservative treatments fail to provide adequate symptom relief [1]. The American College of Rheumatology and the American Association of Hip and Knee Surgeons recommend surgical options like total joint arthroplasty (TJA) for patients with advanced symptomatic OA who do not respond to nonoperative therapies [11]. TJA is highly effective in relieving pain and restoring function, making it a cornerstone in the management of severe OA [7]. However, there are advantages and disadvantages associated with different surgical procedures [12,13], and wear debris-induced osteolysis could induce periprosthetic inflammation and pain resulting in osteoplasty failure in the joints [14].

Indications for surgical procedures include persistent pain, significant functional impairment, and radiographic evidence of joint damage despite exhaustive conservative management [15]. Proper patient selection and timing are crucial to optimize surgical outcomes and enhance postoperative recovery [11]. In this article, we reviewed and critically analyzed the reports in the literature on the advantages and disadvantages of surgical interventions in severe OA.

## 2. Methods

We searched the original research articles, case reports, and selected review articles, such as Cochrane review, systematic review, and meta-analysis, in PubMed and Google Scholar published in years 2005–2025 using the key search terms: Osteoarthritis, total joint arthroplasty, total knee arthroplasty, total hip arthroplasty, Osteotomy, arthroscopy, pain, and surgical interventions. The articles published in English language only were included for initial review of the title and abstract. Book chapters, letters to the editor, commentaries, and other reports irrelevant to the subject were excluded. Relevant reports, including clinical guidelines, were critically reviewed for the goal of the study, methods, key findings, and limitations.

### 2.1 Overview of Surgical Interventions for Severe Osteoarthritis

TJA is a highly effective surgical intervention for patients with severe OA who have not responded to conservative treatments. The American College of Rheumatology and the American Association of Hip and Knee Surgeons recommend TJA for patients with moderate-to-severe pain and functional impairment, supported by radiographic evidence of joint damage. TJA aims to relieve pain, restore function, and improve quality of life [11]. Total Knee Arthroplasty (TKA) is one of the most common procedures for end-stage knee OA. It involves replacing the entire knee joint with a prosthesis. TKA has evolved significantly, with modern designs focusing on improving knee kinematics and reducing wear rates [16]. TKA is indicated for patients with severe knee pain and functional limitations who have exhausted nonoperative therapies [11]. Total Hip Arthroplasty (THA) is the gold standard for treating severe hip OA. It involves replacing the damaged hip joint with a prosthetic implant. THA is highly effective in reducing pain and improving mobility. The American College of Rheumatology emphasizes the importance of patient selection and timing to optimize outcomes [17].

Partial Joint Replacement is an option for patients with OA confined to a single compartment of the joint. This approach preserves more of the natural joint of a patient and can result in faster recovery and fewer complications compared to total joint replacement [18]. However, it may have higher revision rates in the long term [19]. Unicompartmental Knee Arthroplasty (UKA) is a type of partial knee replacement suitable for patients with OA limited to one compartment of the knee [20]. UKA offers benefits such as shorter hospital stays, faster recovery, and better early functional outcomes compared to TKA [21]. However, UKA has a higher risk of revision surgery, particularly for disease progression in other compartments [22].

Osteotomy is a surgical procedure aimed at realigning the joint to redistribute weight and relieve pain in patients with unicompartmental knee OA. It is particularly beneficial for younger, active patients who are not ideal candidates for TKA. The goal is to transfer the load from the damaged compartment to healthier areas of the joint, thereby delaying the need for joint replacement [22]. High Tibial Osteotomy (HTO) is a specific type of osteotomy used to treat medial compartment OA by shifting the weight-bearing axis from the arthritic medial compartment to the lateral compartment. This procedure is indicated for patients with varus deformity and isolated medial compartment OA [7]. The American Academy of Orthopedic Surgeons (AAOS) notes that HTO can improve pain and function in properly selected patients [23]. Femoral osteotomy is used to correct deformities in the femur that contribute to knee OA.

It is often performed in conjunction with other procedures, such as TKA, to address severe extraarticular deformities. This approach can restore mechanical alignment and improve joint function [24].

Arthroscopy involves minimally invasive techniques to diagnose and treat joint issues. However, its role in managing OA is limited. The Cochrane Review indicates that arthroscopic surgery, including debridement and lavage, provides little to no benefit in pain or function for knee OA compared to placebo surgery. The AAOS also recommends against routine use of arthroscopy for primary knee OA [24]. Debridement involves the removal of loose cartilage and other debris from the joint, while lavage flushes out the joint space. These procedures may offer short-term symptom relief but do not alter the disease progression [25]. The American Society of Pain and Neuroscience notes that these interventions should not be used as routine treatments for OA [22].

Joint fusion, or arthrodesis, is a surgical procedure used to achieve bone fusion in a joint, providing pain relief and stability in cases where other treatments have failed. It is often considered a salvage procedure for severe OA, particularly when TJA is not viable due to infection, severe bone loss, or other complications. Arthrodesis is indicated in patients with severe OA who have failed previous surgical interventions, such as TJA, or in cases of chronic infection, post-traumatic arthritis, and periarticular tumors. The primary goal is to provide a stable, pain-free joint. Studies have shown that knee arthrodesis can achieve solid fusion in 65–75% of cases, with intramedullary nail fixation often yielding higher fusion rates compared to external fixation [26]. However, complications such as nonunion, infection, and the need for repeat surgeries are common [27].

Recent advancements in surgical techniques for arthrodesis include the use of intramedullary nails, compression plates, and the Ilizarov circular external fixator. Intramedullary nailing has shown superior outcomes in terms of fusion rates and stability, particularly in the presence of severe bone loss [28]. The Ilizarov technique, although less commonly used, provides rigid fixation and high fusion rates, especially in cases with persistent infection [29]. Emerging biologic treatments and cartilage restoration procedures aim to repair and regenerate damaged cartilage, potentially delaying or obviating the need for arthrodesis. Techniques such as autologous chondrocyte implantation, osteochondral autograft transplantation, and the use of growth factors and stem cells are being explored. These procedures are still under investigation, and their long-term efficacy in severe OA remains to be fully established.

## 3. Advantages of Surgical Interventions

### 3.1 Pain Relief and Functional Improvement

Surgical interventions, particularly TJA are highly effective in providing significant pain relief and functional improvement for patients with severe osteoarthritis. Clinical studies have consistently demonstrated the benefits of these procedures.

Total knee and hip arthroplasty are among the most successful surgical procedures for severe OA, offering substantial pain relief and restoration of function. According to a review in Osteoarthritis and Cartilage, TJA significantly reduces pain and improves quality of life in patients with advanced OA [30]. A longitudinal study published in BMC Musculoskeletal Disorders found that patients undergoing total hip or knee replacement reported significant improvements in pain and physical function, as measured by the Western Ontario and McMaster Universities Osteoarthritis Index and the WHOQOL-BREF physical domain [7]. Clinical trials have shown that surgical interventions like TKA lead to marked reductions in pain. For instance, a study in The Journal of Bone and Joint Surgery reported that patients with severe preoperative pain experienced significant pain relief one and two years postoperatively [31]. Another study in Plastic and Reconstructive Surgery highlighted the efficacy of surgical knee denervation in reducing pain for patients who were not candidates for TKA, with a decrease in visual analogue scale pain scores from 8.7 to 2.9 [32]. Surgical interventions also enhance mobility and the ability to perform daily activities. The same longitudinal study in BMC Musculoskeletal Disorders noted significant improvements in physical function domains post-surgery, particularly in younger patients and those with manual jobs [30]. Additionally, a network meta-analysis in BMC Musculoskeletal Disorders indicated that total knee arthroplasty and unicompartmental knee arthroplasty are superior in reducing complications and improving functional outcomes compared to other surgical options [33].

### 3.2 Long-term Benefits

Total joint replacements, such as TKA and THA, have been shown to provide substantial long-term benefits. Studies indicate that these procedures result in significant improvements in pain relief, functional status, and overall quality of life for up to 10 years postoperatively [34]. For instance, a systematic review and meta-analysis demonstrated that TKA leads to marked improvements in pain and function, with most patients reporting high levels of satisfaction [35]. The durability of joint replacements is a critical factor in their success. Research shows that approximately 82% of TKAs and 58% of THAs last at least 25 years, making them reliable longterm solutions for severe OA. Additionally, specific designs, such as the non-modular InsallBurstein I component, have shown superior long-term survivorship compared to other designs [36]. This durability ensures that patients can maintain improved joint function and reduced pain over extended periods [37]. Surgical interventions significantly enhance the quality of life for patients with severe OA. Total joint replacements have been associated with substantial improvements in both disease-specific and generic health-related quality of life measures. For example, patients undergoing TKA or THA report significant gains in physical health, social relationships, and overall well-being [30]. These improvements are maintained for several years post-surgery, highlighting the profound impact of these procedures on patients’ lives [34].

### 3.3 Technological Advancements

Technological advancements in surgical interventions for severe osteoarthritis have significantly improved patient outcomes. The introduction of computer navigation and robotic assisted systems in TKA has enhanced the precision of component placement and alignment, leading to better functional outcomes and reduced complications. Studies have shown that computer-navigated TKA is associated with lower risks of periprosthetic joint infection, pulmonary embolism, and acute respiratory failure compared to traditional TKA. Similarly, robotic-assisted TKA has demonstrated lower risks of deep vein thrombosis, myocardial infarction, and pulmonary embolism [38].

Minimally invasive approaches in joint replacement surgery have also gained popularity due to their potential benefits, including reduced postoperative pain, shorter hospital stays, and faster recovery times. These techniques involve smaller incisions and less soft tissue disruption, which can lead to improved cosmetic outcomes and decreased risk of infection [39]. The use of image-guided orthopedic surgery (IGOS) has further enhanced the accuracy of these minimally invasive procedures, allowing for precise surgical planning and real-time navigation during the operation [40].

Robotics and navigation in joint replacement have revolutionized the field by providing surgeons with advanced tools to achieve optimal outcomes. Robotic systems, such as robotic arm-assisted TKA, offer improved accuracy in component placement and alignment, which can lead to better long-term implant survivorship and patient satisfaction [41]. These systems utilize preoperative planning and intraoperative guidance to ensure precise execution of the surgical plan, reducing variability and increasing consistency in outcomes [41]. Additionally, robotic-assisted surgeries have been shown to decrease postoperative complications and opioid consumption, further enhancing patient recovery [38].

### 3.4 Cost-Effectiveness in the Long Run

**Figure 1:**
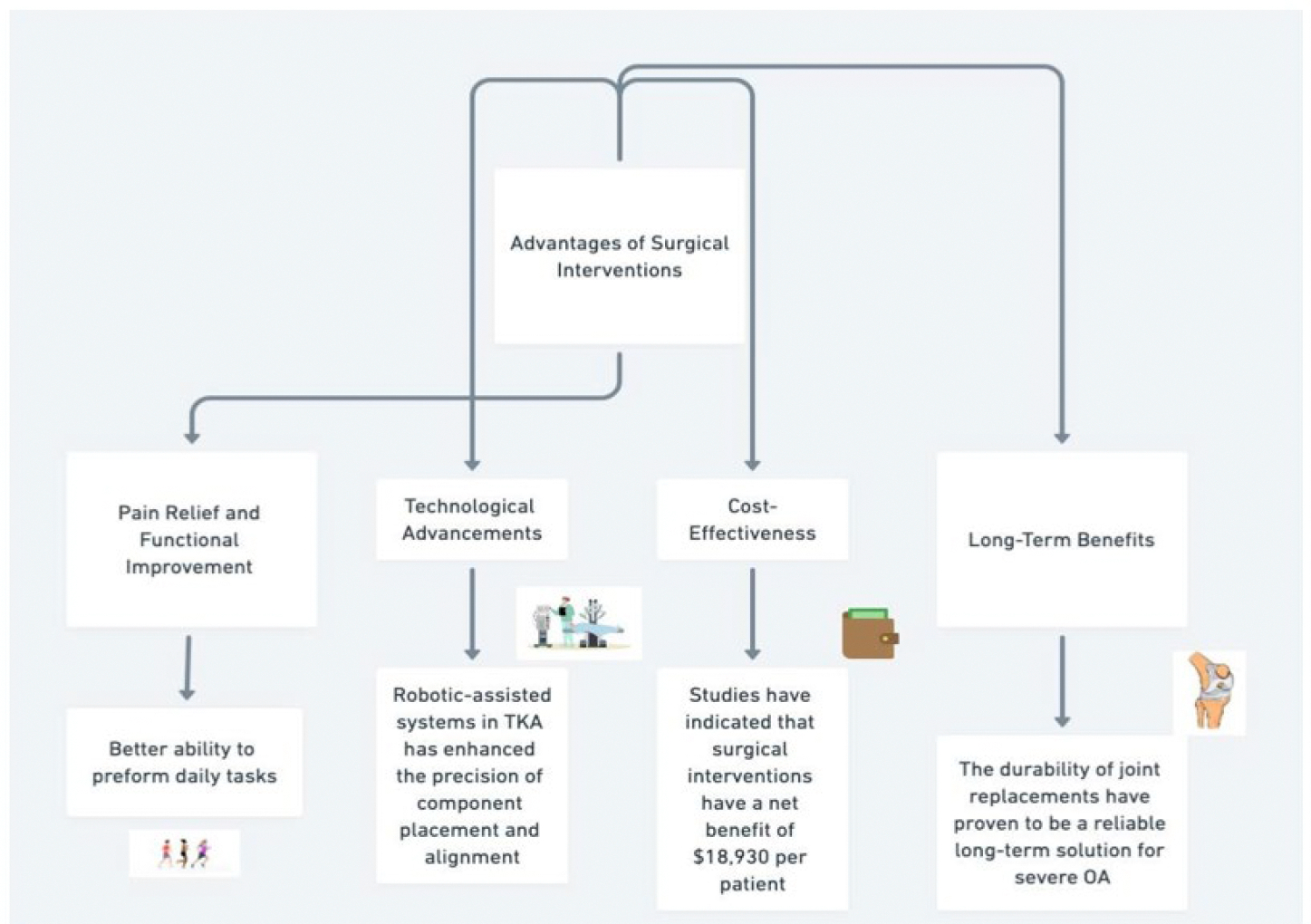
Illustrates the advantages of surgical interventions in the treatment of severe osteoarthritis (OA). TKA, total knee arthroplasty.

Surgical interventions for severe osteoarthritis, particularly TKA and UKA, have been shown to be cost-effective over a patient’s lifetime. A study using a Markov decision analytic model found that surgical treatments for unicompartmental knee osteoarthritis were less expensive and provided more quality-adjusted life years compared to nonsurgical treatments for patients aged 40 to 69 years [42]. Additionally, the societal savings from the preferential use of UKA over TKA were estimated to be between $987 million to $1.5 billion per annual wave of patients undergoing treatment (Figure 1). Another study highlighted that TKA increased lifetime direct costs by a mean of $20,635 but resulted in societal savings of $39,565 from reduced indirect costs, leading to a net benefit of $18,930 per patient [43].

Surgical interventions significantly reduce the healthcare burden associated with chronic pain management in severe osteoarthritis. By effectively alleviating pain and improving function, procedures like TKA and UKA decrease the need for long-term pharmacotherapy and frequent medical consultations. This reduction in chronic pain management not only improves patient quality of life but also translates into substantial healthcare cost savings [43]. For instance, the societal savings from TKA, primarily due to increased employment and earnings, were estimated to be approximately $12 billion from the more than 600,000 procedures performed in the U.S. in 2009. Furthermore, a systematic review confirmed that TKA and THA are cost-effective and improve quality of life compared to non-operative treatments [43].

## 4. Disadvantages and Risks of Surgical Interventions

### 4.1 Surgical Risks and Complications

Surgical interventions for severe osteoarthritis, while effective in alleviating symptoms and improving function, carry several disadvantages related to surgical risks and complications. Surgical procedures such as TKA and THA are associated with various risks, including component failure, surgical site infection, knee stiffness, and deep vein thrombosis (DVT) [43]. Outpatient TKA, for instance, has been linked to higher rates of perioperative complications compared to inpatient procedures. Additionally, the surgical approach can influence complication rates, with the anterior approach in THA showing higher complication rates compared to the posterior approach [44].

Postoperative infections, both superficial and deep, are significant concerns. Obese patients undergoing THA have a higher risk of deep infections, which can lead to prolonged hospital stays and increased healthcare costs [45]. Infection rates are also higher in patients with posttraumatic arthritis compared to those with osteoarthritis [46].

Surgical interventions carry risks of bleeding and complications related to anesthesia. Factors such as patient age, comorbidities, and the complexity of the procedure can increase these risks [47]. Anesthesia-related complications, although less common, can include respiratory issues and adverse reactions to anesthetic agents [48].

The risk of DVT and pulmonary embolism (PE) is a well-documented complication following knee and hip arthroplasty [25]. Prophylactic measures, such as the use of low-molecular-weight heparin, are recommended to mitigate these risks [49]. Despite these measures, the incidence of DVT and PE remains a concern, particularly in high-risk patient populations [50].

### 4.2 Implant Failure and Revision Surgeries

Implant failure is a significant concern following TKA and THA, often necessitating revision surgeries. The primary causes of implant failure include aseptic loosening, instability, and periprosthetic infection. Aseptic loosening, which results from chronic inflammation due to wear debris, remains a leading cause of revision surgeries. Instability and malalignment are also common, with instability accounting for approximately 14.1% of TKA revisions [51]. Periprosthetic infections, which can be challenging to treat, are increasingly recognized as a major cause of implant failure, contributing to 21.6% of TKA revisions [52].

Loosening and wear of the implant components are critical issues that can lead to pain, functional impairment, and the need for revision surgery. Polyethylene wear, although less common now due to improved materials, still occurs and can lead to osteolysis and aseptic loosening. Dislocation is another complication, particularly in THA, where it accounts for 15.9% of revisions. The American College of Radiology notes that instability and malalignment can exacerbate these issues, leading to further complications and the need for additional surgical interventions [53].

Revision surgeries are inherently more complex and carry higher risks compared to primary arthroplasties. The technical challenges include managing bone loss, ensuring proper alignment, and achieving stable fixation of the new implant. Patients undergoing revision surgeries often have poorer outcomes and higher complication rates, including increased risk of infection and mechanical failure [54]. The American Physical Therapy Association highlights that complications such as periprosthetic lucency, subluxation, and erosion are common in revision surgeries, often requiring further interventions. Additionally, patients with comorbid conditions like osteoporosis are at higher risk for complications such as periprosthetic fractures and aseptic loosening, further complicating revision procedures [55].

### 4.3 Postoperative Rehabilitation and Recovery Challenges

The postoperative period presents several challenges that must be addressed to optimize outcomes. Postoperative rehabilitation is crucial for successful recovery after TJA. Challenges include managing pain, inflammation, and cognitive dysfunction, which can affect physical and cognitive function post-surgery. Effective multimodal opioid-sparing analgesic regimens and patient-specific physiotherapy programs are essential to address these issues [56].

Recovery time after TJA can vary, but significant improvements in physical and mental impairments are typically observed within the first three months postoperatively [57]. Full recovery, including participation in social and work activities, may take up to 12 months. Early mobilization and adherence to rehabilitation protocols are critical for expediting recovery [58].

Physical therapy is a cornerstone of postoperative care, with evidence supporting its role in enhancing recovery and functional outcomes. The American Physical Therapy Association recommends starting physical therapy within 24 hours of surgery and continuing it post discharge to ensure optimal recovery. However, compliance with physical therapy can be challenging due to factors such as pain, logistical issues, and patient motivation. Strategies like health coaching and financial incentives have been shown to improve physical activity and compliance with physical therapy [59].

### 4.4 Comparison of Outcomes: Surgical vs. Non-Surgical Management

#### Effectiveness of Conservative Management

Conservative management of OA, including physical therapy, weight loss, and lifestyle modifications, is often the first line of treatment. The American College of Rheumatology/Arthritis Foundation recommends weight loss for overweight or obese patients with knee and/or hip OA, as it can significantly improve symptoms and function [60]. Exercise, including land-based and aquatic, has been shown to improve pain and physical function in patients with knee OA [61].

Nonsteroidal anti-inflammatory drugs (NSAIDs) are commonly used for pain management in OA. Both oral and topical NSAIDs are effective in reducing pain and improving function, although they carry risks, particularly in patients with comorbidities [11]. Intra-articular corticosteroid injections provide short-term pain relief and are recommended for managing OA pain [1]. Duloxetine is conditionally recommended for patients with knee, hip, and/or hand OA due to its efficacy in pain management [60].

Weight loss is strongly recommended for patients with knee and/or hip OA who are overweight or obese. A loss of ≥5% of body weight can lead to significant improvements in symptoms and function, with greater benefits observed with higher weight loss [60]. Lifestyle modifications, including dietary changes and increased physical activity, are essential components of OA management.

Surgical interventions, such as TJA, are considered when conservative treatments fail to provide adequate relief. TJA is highly effective in relieving pain and restoring function in patients with advanced OA [1]. However, non-surgical management can delay or reduce the need for surgery [62]. Studies have shown that multidisciplinary non-operative management can lead to significant improvements in symptoms and function, potentially avoiding the need for surgery [15].

#### Patient Selection for Surgery

Patient selection for surgical intervention in OA is critical to achieving optimal outcomes. The American College of Rheumatology and the American Association of Hip and Knee Surgeons recommend TJA for patients with symptomatic moderate-to-severe OA who have not responded to nonoperative therapies such as physical therapy, NSAIDs, and intraarticular injections [63,64]. Factors such as radiographic severity, functional impairment, and pain levels are key determinants in selecting patients for surgery [65].

Several factors influence the decision to proceed with surgery, including the severity of symptoms, the patient’s overall health, and their expectations from the surgery. Patients with severe pain, significant functional limitations, and poor response to conservative treatments are more likely to be considered for surgery [66]. Psychological factors, such as depression and anxiety, also play a role in decision-making, as patients with worse psychological profiles may have higher expectations from surgery [67].

Patient-reported outcomes and satisfaction are essential metrics for evaluating the success of surgical interventions. Studies have shown that TJA significantly improves pain, function, and quality of life in patients with severe OA [68]. However, patient satisfaction is influenced by preoperative expectations and the extent of functional improvement achieved postoperatively [69]. Non-surgical management, while effective in delaying surgery, often results in poorer long-term outcomes compared to surgical interventions [69].

## 5. Future Directions and Innovations in Surgical Management of OA

Severe osteoarthritis often necessitates surgical intervention when conservative treatments fail to provide adequate relief. The primary surgical option for end-stage OA is total joint arthroplasty, which offers significant pain relief and functional improvement [7]. However, advancements in biologic and regenerative therapies, such as stem cell therapy and platelet-rich plasma, are gaining traction for their potential to enhance tissue regeneration and delay the need for joint replacement [70]. These therapies aim to harness the body’s natural healing processes to repair damaged cartilage and reduce inflammation [70].

Improved implant materials and designs have also revolutionized surgical outcomes. Modern implants are designed to mimic natural joint mechanics more closely, thereby improving longevity and reducing wear [71]. Enhanced rehabilitation protocols, including personalized and AI-guided approaches, are being integrated to optimize recovery and functional outcomes post-surgery [70]. These protocols are tailored to individual patient needs, leveraging data analytics and machine learning to predict recovery trajectories and customize rehabilitation plans.

Personalized medicine and AI-guided surgical approaches are at the forefront of orthopedic surgery innovation. These technologies enable more precise surgical planning and execution, potentially reducing complications and improving patient-specific outcomes [70]. The integration of these advanced techniques and materials represents a significant shift towards more effective and individualized treatment strategies for severe OA, aiming to improve both short-term recovery and long-term joint function.

## 6. Conclusion

Surgical interventions for severe osteoarthritis play a crucial role in restoring function and alleviating pain when conservative treatments fail. Total joint arthroplasty, including total knee arthroplasty and total hip arthroplasty, remains the gold standard, offering long-term benefits such as improved mobility, pain relief, and enhanced quality of life. However, alternative procedures, such as partial joint replacement, osteotomy, and arthrodesis, provide tailored solutions for select patient populations. Despite their effectiveness, surgical procedures come with inherent risks, including perioperative complications, implant failure, and the need for revision surgeries. Additionally, the success of surgical outcomes relies heavily on proper patient selection, adherence to rehabilitation protocols, and technological advancements in surgical techniques, such as robotic-assisted procedures and personalized medicine.

While non-surgical management remains viable for delaying surgery and improving symptoms, its long-term efficacy is limited for patients with advanced disease. Emerging regenerative therapies, including stem cell treatments and platelet-rich plasma injections, hold promise in reducing inflammation and potentially postponing the need for surgery. Ultimately, the **decision** to undergo surgical intervention must be individualized, balancing the benefits against potential risks. Advancements in biomaterials, surgical precision, and postoperative rehabilitation strategies continue to shape the future of osteoarthritis management, aiming to optimize patient outcomes and improve long-term joint function.

## Key points

- Osteoarthritis is the most common form of arthritis, characterized by progressive joint degeneration leading to pain and functional impairment.
- In cases where total joint arthroplasty is not viable, arthrodesis may be considered to achieve pain relief by permanently fusing the affected joint.
- Arthroscopy procedures such as lavage and debridement have limited benefits in osteoarthritis management and are not recommended for routine treatment.
- High tibial osteotomy is used to realign the knee joint in patients with medial compartment osteoarthritis, thereby redistributing weight to relieve pain.
- Severe osteoarthritis often necessitates surgical intervention when conservative treatments fail to provide adequate symptom relief.
- Osteotomy is a joint-preserving surgical technique that redistributes mechanical load in patients with unicompartmental knee osteoarthritis.
- Total joint arthroplasty remains the most effective surgical option for relieving pain and restoring function in patients with severe osteoarthritis.
- Surgical interventions, including total joint arthroplasty and osteotomies, are critical options for patients who do not respond to nonoperative management.
- Total hip arthroplasty is the gold standard for managing severe hip osteoarthritis, significantly improving mobility and reducing pain.
- Total joint arthroplasty, including knee and hip replacements, is recommended for patients with advanced osteoarthritis to improve quality of life.
